# Red cell distribution width is associated with long-term prognosis in patients with stable coronary artery disease

**DOI:** 10.1186/1471-2261-13-113

**Published:** 2013-12-10

**Authors:** Tadeusz Osadnik, Joanna Strzelczyk, Michał Hawranek, Andrzej Lekston, Jarosław Wasilewski, Anna Kurek, Aleksander Rafał Gutowski, Krzysztof Wilczek, Krzysztof Dyrbuś, Marek Gierlotka, Andrzej Wiczkowski, Mariusz Gąsior, Andrzej Szafranek, Lech Poloński

**Affiliations:** 1 IIIrd Chair and Department of Cardiology, Silesian Centre for Heart Diseases, Medical University of Silesia in Katowice, Medical Faculty in Zabrze, Ul. Marii Skłodowskiej Curie 9, 41-800, Zabrze, Poland; 2 Department of Medical Biology, Medical University of Silesia in Katowice, Medical Faculty in Zabrze, Ul. Marii Skłodowskiej Curie 9, 41-800, Zabrze, Poland

**Keywords:** Red cell distribution width, Stable coronary artery disease, Prognosis

## Abstract

**Background:**

Data regarding the association between red cell distribution width (RDW) values and mortality in patients with stable coronary artery disease are scarce. We aimed to investigate the link between mortality and RDW in patients with stable coronary artery disease undergoing percutaneous coronary intervention (PCI).

**Methods:**

We analyzed 2550 consecutive patients with stable coronary artery disease who underwent PCI between 2007 and 2011 at our institution. The patients were divided into four groups according to RDW quartiles. The association between the RDW values and the outcomes was assessed using Cox proportional regression analysis after adjusting for clinical, echocardiographic, hemodynamic and laboratory data in the whole population and in subgroups stratified by gender, presence of diabetes, anemia or heart failure.

**Results:**

In the entire population, there was a stepwise relationship between RDW intervals and comorbidities. Patients with the highest RDW values were older and more often burdened with diabetes, heart failure and chronic kidney disease. There was an almost 4-fold increase in mortality during an average of 2.5 years of follow-up between the group of patients with RDW values lower than 13.1% (25^th^ percentile) and the group with RDW values higher than 14.1% (75^th^ percentile), (4.3% vs. 17.1%, p < 0.0001). After adjusting for the covariates, RDW remained significantly associated with mortality in the whole cohort (HR-1.23 [95% CI (1.13-1.35), p < 0.0001]) and in the subgroups stratified by gender, age (over and under 75 years), presence of anemia, diabetes, heart failure and chronic kidney disease.

**Conclusion:**

Higher RDW values correspond to higher comorbidity burdens and higher mortality. RDW is an independent predictor of mortality in patients with stable coronary artery disease.

## Background

Red cell distribution width (RDW) is a numerical measure of erythrocyte variability and heterogeneity (i.e., anisocytosis). RDW is elevated in patients with anemia or thalassemia and after a blood transfusion or in the presence of iron deficiency [1]. The upper and lower limits of the RDW values were set at the 5^th^ (11.0%) and 95^th^ (14.0%) percentiles in a population from a National Health and Nutrition Examination Survey III study. In recent years there have been studies referring to patients with heart failure [2], with acute coronary syndromes [3] and unselected patients undergoing percutaneous coronary intervention (PCI) [4,5] and various non-cardiological conditions [6-8]. To the best of our knowledge, there was only one smaller study referring to consecutive patients with stable coronary artery disease undergoing elective stent implantation with eight registered deaths in the 12 months follow-up [9], and two referring to subsets of patients with stable coronary artery disease [10,11]. We aimed to investigate the link between mortality and RDW in the wide spectrum of patients with stable coronary artery disease undergoing PCI with stent implantation over long term follow-up.

## Methods

### Study group

Data from consecutive patients with stable coronary artery disease undergoing stent implantation between 2007 and 2011 at our institution (the Silesian Center for Heart Diseases) were analyzed. To identify patients with stable coronary artery disease, we screened all patients with the diagnosis codes of I25.0 and I25.2, as well as patients with other diagnoses who met the following criteria: i) elective hospital admission and ii) stent implantation. Patients undergoing concomitant transcatheter aortic valve implantation procedure, patients undergoing hybrid revascularization, and patients after orthotropic heart transplant were not considered in the first place. We have identified 2774 patients with stable coronary artery disease. Patients who died during hospitalization (n = 4), patients on dialysis (n = 11), those with advanced valve disease (n = 203), a history of cancer (n = 26) or other diseases potentially limiting survival (n = 18) were excluded from the analysis. Final cohort consisted of 2550 patients.

### Data source

Starting in 2006, it has been compulsory for every attending physician at our Institution to fill out a complex report form for all admitted patients. This report form includes clinical data, past medical history and performed procedures. The form includes detailed data on a patient’s medical history and clinical characteristics at admission, and it resembles the cardiac report form used in clinical studies. Before patient documentation is given to the hospital information archive, the course of hospitalization is entered, and the report form is checked for completeness. Despite these strict measures, 11 (0.4%) patients were found to have missing data regarding information on the family history (FH) of premature coronary heart disease (CHD), Canadian Cardiovascular Society class, heart rate or systolic blood pressure (SBP) at admission. Data on ejection fraction were available for 2322 (91.1%) patients. Hemodynamic data were available for all patients and were taken from angiography descriptions. Creatinine, sodium levels and complete blood counts were available for all patients. The complete blood counts were performed using the Sysmex XS1000i and XE2100 (Sysmex Corporation, Kobe, Japan). Red cell distribution width (RDW) is calculated using the following formula: RDW = (standard deviation of red blood cell corpuscular volume)/(mean corpuscular volume (MCV)) × 100 [%]. The creation of the database of patients with stable coronary artery disease used in this study was supported by the National Science Center – Dec-2011/01/D/NZ5/04387. Study was approved by ethics committee at district chamber of physicians.

### Statistical analysis

The continuous variables are presented as the means and standard deviations. The categorical variables are presented as percentages. Patients were divided into subgroups according to RDW quartiles. Group I (n = 607) comprised patients with an RDW < 13.1% (1^st^ quartile), group II (n = 574) comprised patients with an RDW value of ≥13.1% and < 13.6% (2^nd^ quartile), group III (n = 663) comprised patients with RDW values of ≥ 13.6% and <14.1% (3^rd^ quartile) and group IV (n = 706) comprised patients with RDW values ≥ 14.1% (4^th^ quartile). To test for differences across all groups, the chi square and Kruskall-Wallis tests were used.

### Survival and regression analysis

The associations between the RDW quartiles and mortality were analyzed using the Kaplan-Meier method with log-rank testing. To assess the impact of the RDW on prognosis, a multivariate Cox regression analysis was performed. RDW was imputed as a continuous variable. To minimize the impact of missing data on the Cox regression analysis, the multiple imputation method was used to impute missing data for the variables that were to be included in the Cox regression procedures. The model was adjusted for age, sex, heart failure, atrial fibrillation, hypertension, previous myocardial infarction (MI), previous PCI, previous coronary artery bypass graft surgery (CABG), previous sudden cardiac death (SCD), peripheral vascular disease (PVD), previous stroke, diabetes, lipid abnormalities, obesity, chronic obstructive pulmonary disease (COPD), chronic kidney disease (CKD), smoking, New York Heart Association (NYHA) and CCS class, heart rate, blood pressure, ejection fraction, number and type(s) of stent implanted, number of PCI vessels, hemoglobin, MCV. Additionally stratified analyses according to gender, age (over or under 75 years) diabetes, CKD, anemia, and heart failure status were performed. Difference in Cox-model including aforementioned variables was compared with Cox model including additionally RDW using likelihood ratio test. Discriminative ability of those two models was assessed using Harrell’s C-statistics [12]. We have also compared those models by means of Net Reclassification Improvement (NRI) and Integrated Discrimination Improvement (IDI). NRI estimates if the addition of new variable correctly increases or decreases the predicted risk for events and non-events [13-16]. We have calculated NRI using predicted probabilities estimated by Cox regression analysis at the end of follow-up time. IDI describes differences in integrated sensitivity and integrated one minus specificity between models [13].

### Follow-up data

Information on survival was based on the National Health Fund (NFZ) insurance status, which can be electronically verified. Because the NFZ insurance policy is obligatory for all Polish citizens, patients who were insured were marked as alive. We made an attempt to contact the relatives of uninsured patients and/or the relevant local registry office to obtain the exact date of death. Follow-up data were available for 2535 (99.4%) of patients. The mean follow-up period was 915.4 ± 525.3 days. During the observation period, there were 233 reported deaths. All reported p-values are two-sided. The analyses were performed using Number Crunching Statistical Systems 8.0 (NCSS, Kaysville, UT, USA) and in R software [17].

## Results

### Baseline characteristics of the entire population

The baseline clinical characteristics for the entire population are shown in Table 1. In general, patients with the highest RDW values were older and more often burdened with diabetes, heart failure, CKD, COPD and PVD. Moreover, patients with higher RDW values had higher heart rates and lower ejection fractions. Not surprisingly, the lowest hemoglobin values were observed in patients with the highest RDW values (Table 2). The prescribed treatments at discharge reflected differences in comorbidities: more nitrates and diuretics were prescribed in patients with the highest RDW values (Table 3).

**Table 1:** Baseline clinical characteristics by quartiles of RDW

	**Group I**	**Group II**	**Group III**	**Group IV**	**P value**
	**N = 607**	**N = 574**	**N = 663**	**N = 706**	
	**<13.1%**	**13.1 ≥ and <13.6%**	**13.6% ≥ and <14.1%**	**≥14.1**	
Age (yrs)	62.3 ± 9.5	63.3 ± 9.4	64.4 ± 9.3	65.6 ± 9.1	<0.0001
Men (%)	436 (71.8)	408 (71.1)	461 (69.5)	494 (70.0)	0.8
Heart failure	67 (11.0)	69 (12.0)	77 (11.7)	146 (20.7)	<0.0001
Atrial fibrillation	39 (6.4%)	50 (8.7%)	75 (11.3%)	123 (17.4)	<0.0001
Hypertension	428 (70.5)	431 (75.1)	477 (71.9)	491 (69.5)	0.15
Previous MI	343 (56.5%)	320 (55.7%)	373 (56.3%)	437 (61.9%)	0.07
Previous CABG	69 (11.4)	83 (14.5)	89 (13.4)	99 (14.0)	0.38
Previous PCI	307 (50.6)	274 (47.7)	306 (46.2)	367 (52.0)	0.08
Previous SCD	10 (1.6%)	20 (3.5)	21 (3.2)	26 (3.7)	0.14
PVD	24 (4.0)	34 (5.9)	37 (5.6)	47 (6.7)	0.19
Prev. Stroke/TIA	19 (3.1)	27 (4.7)	39 (5.9)	60 (8.5)	0.0003
CKD GFR < 60 ml/h/1.73 m^2^	40 (6.6)	59 (10.3)	91 (13.7)	137 (19.4)	<0.0001
Diabetes	210 (34.6)	215 (37.5)	238 (35.9)	280 (39.7)	0.26
Insulin	61 (10.0)	86 (15.0)	85 (12.8)	127 (18.0)	0.0004
Lipid abnormalities	363 (59.8)	357 (62.2)	387 (58.4)	353 (50.0)	<0.0001
Obesity	203 (33.4)	192 (33.4)	234 (35.3)	252 (35.7)	0.75
COPD	32 (5.3)	35 (6.1)	45 (6.8)	79 (11.2)	0.0001
Current smoker	59 (9.7)	65 (11.3)	73 (11.0)	85 (12.0)	0.68
Previous smoker	202 (33.3%)	196 (34.1)	226 (34.1)	259 (36.7)	0.67
FH of premature CHD	65 (10.7)	48 (8.4)	70 (10.6)	47 (6.7)	0.11
NYHA class III/IV	28 (4.6%)	40 (7.0%)	33 (5.0%)	86 (12.2%)	<0.0001
Disabling angina (CCS III/IV)	97 (16.0)	92 (16.0)	115 (17.3)	115 (16.3)	0.9
HR (1/min)	69.3 ± 10.9	70.2 ± 11.4	71.9 ± 11.8	73.0 ± 12.9	<0.0001
SBP (mmHg)	133± 19.7	133.0 ± 19.7	132.3 ± 18.5	132 ± 20.6	0.65
DBP (mmHg)	79.3 ± 10.8	78.9 ± 11.3	78.8 ± 10.6	78.4 ± 12.0	0.20
Sinus rhythm -	588 (96.9)	547 (95.3)	620 (93.5)	632 (89.5)	<0.0001
Ejection fraction (%)	46.7 ± 9.7	47.8 ± 9.48	46.4 ± 10.0	43.8 ± 11.4	<0.0001
BMS	253 (41.7%)	284 (49.5%)	387 (58.4%)	435 (61.6%)	<0.0001
MVD	68 (11.2)	92 (16.0)	106 (16.0)	113 (16.0)	0.04
No. of PCI vessels	1.2 ± 0.5	1.2 ± 0.5	1.2 ± 0.5	1.3 ± 05	0.82
No. of stents	1.4 ± 0.7	1.4 ± 0.7	1.3 ± 0.6	1.4 ± 0.7	0.41

MI – myocardial infarction, CABG – coronary artery bypass graft, PCI – percutaneous coronary intervention, SCD – sudden cardiac death, PVD – peripheral vascular disease, FH – family history, CKD – chronic kidney disease, CHD – coronary heart disease, NYHA – New York Heart Association class, CCS – Canadian Cardiovascular Society class, HR – heart rate, SBP – systolic blood pressure, DBP – diastolic blood pressure, BMS – bare metal stent, MVD – multivessel disease.

**Table 2:** Laboratory findings by quartiles of RDW

	**Group I**	**Group II**	**Group III**	**Group IV**	**P value**
	**N = 607**	**N = 574**	**N = 663**	**N = 706**	
	**<13.1%**	**13.1 ≥ and <13.6%**	**13.6% ≥ and <14.1%**	**≥14.1 %**	
RBC (mln/mm^3^)	4.5 ± 0.4	4.5 ± 0.5	4.5 ± 0.5	4.5 ± 0.5	0.02
HGB (mmol/l)	8.7 ± 0.8	8.6 ± 0.9	8.6 ± 0.8	8.3 ± 1.0	<0.0001
HCT (%)	40.4 ± 3.4	40.6 ± 4.3	40.8 ± 3.8	39.8 ± 4.4	<0.0001
MCV (mm^3^)	90.6 ± 3.9	89.0 ± 3.8	90.5 ± 4.3	89.9 ± 5.6	0.01
RDW (%)	12.6 ± 0.4	13.3 ± 0.1	13.8 ± 0.2	15.0 ± 1.2	<0.0001
WBC (tys/mm^3^)	7.1 ± 1.9	7.3 ± 2.0	7.4 ± 2.6	7.4 ± 2.2	0.17
PLT (tys/mm^3^)	211.3 ± 51.3	210.0 ± 59.4	207.4 ± 60.6	213.3 ± 73.7	0.19
Creatinine (μmol/l)	79.8 ± 32.6	80,3 ± 22.8	83,2 ± 24.7	89,8 ± 48.1	<0.0001
GFR (ml/min/1.73 m^2^)	90.0 ± 21.9	89.4 ± 25.5	85.5 ± 24.2	82,4 ± 26.5	<0.0001
Sodium (mmol/l)	138.5 ± 2.6	138.3 ± 2.8	138.7 ± 2.64	138.5 ± 2.8	0.09
Glucose (mmol/l)	6.2 ± 2.0	6.1 ± 2.0	6.0 ± 1.6	5.9 ± 1.7	0.18
Cholesterol (mmol/l)	4.3 ± 1.1	4.5 ± 1.2	4.4 ± 1.1	4.5 ± 1.3	0.08

RBC – red blood cells, HGB – hemoglobin, HCT – hematocrit, MCV – mean corpuscular volume, RDW – red cell distribution width, WBC – white blood cells, PLT – platelets, GFR – glomerular filtration rate.

**Table 3:** Prescribed treatment at discharge by quartiles of RDW

	**Group I**	**Group II**	**Group III**	**Group IV**	**P value**
	**N = 607**	**N = 574**	**N = 663**	**N = 706**	
	**<13.1%**	**13.1 ≥ and <13.6%**	**13.6% ≥ and <14.1%**	**≥14.1%**	
Aspirin	601 (99.0)	569 (99.1)	658 (99.2)	693 (98.2)	0.21
Clopidogrel/Thienopiridines	607 (100)	572 (99.7)	658 (99.2)	699 (99.0)	0.25
ACE-I/ARB	585 (96.4)	539 (93.9)	622 (93.8)	641 (90.8)	0.0006
Beta-blockers	581 (95.7)	555 (96.7)	630 (95.0)	671 (95.0)	0.44
Calcium channel blockers	106 (17.5)	82 (14.3)	100 (15.1)	112 (15.9)	0.47
Diuretics	203 (33.4)	194 (33.8)	229 (34.5)	315 (44.6)	<0.0001
Nitrates	121 (19.9)	166 (28.9)	223 (33.6)	233 (33.0)	<0.0001
VKA	23 (3.8)	32 (5.6)	47 (7.1)	83 (11.8)	<0.0001

ACE-I – angiotensin converting enzyme inhibitors, ARB – angiotensin receptor blockers, VKA – vitamin K antagonists.

### Survival analysis and predictors of outcome

**Figure 1:**
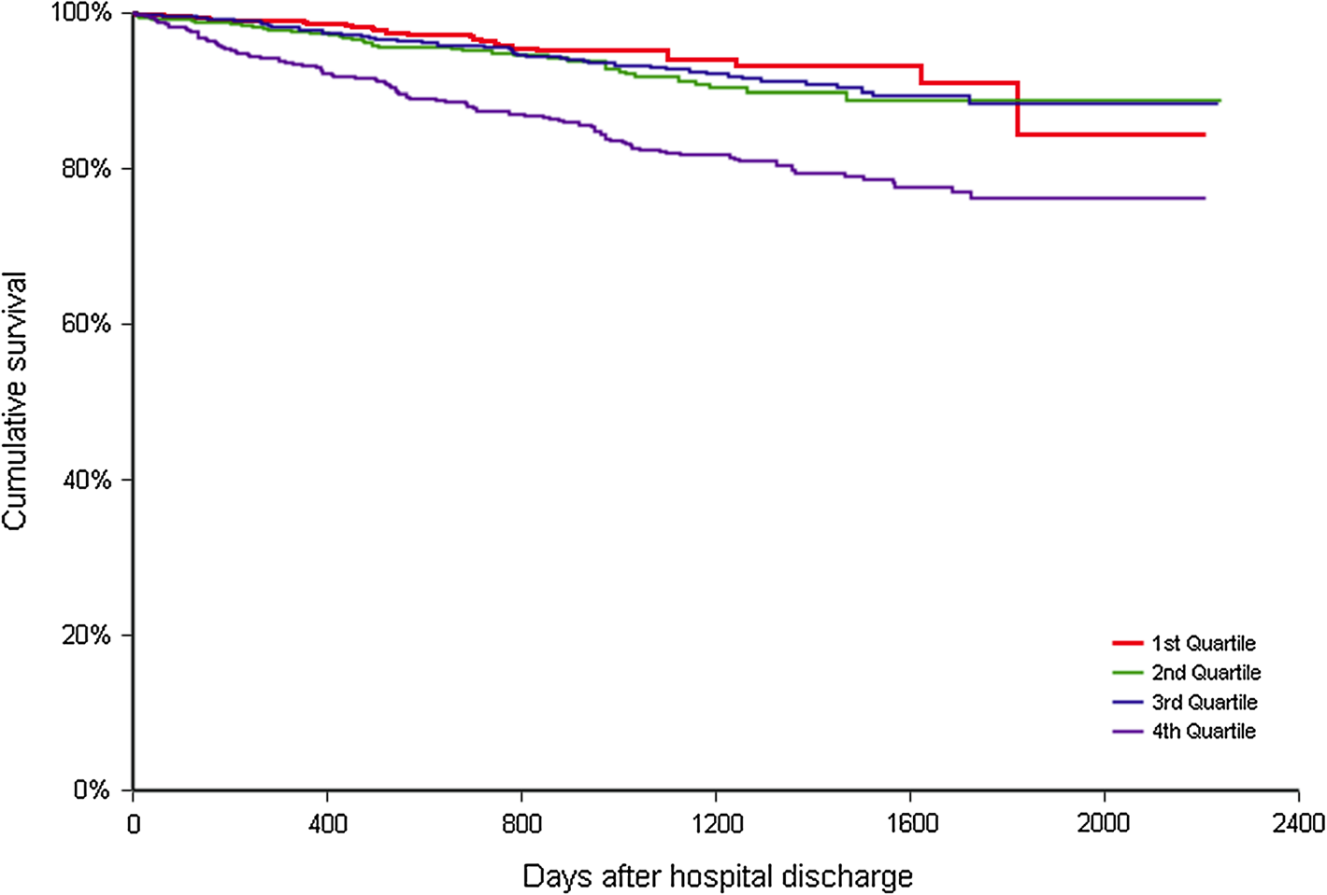
Kaplan Meier survival curves by quartiles of RDW.

During the observation period, there were 233 reported deaths. Mortality in quartiles 1 to 3 was significantly lower as compared with quartile 4 (quartile 1 – 26 (4.3%); quartile 2 – 39 (6.8%); quartile 3–48 (7.3%); quartile 4–120 (17.1%), p < 0.0001, respectively) Figure 1. RDW was highly significantly associated with mortality in the entire cohort and in a subset of patients stratified by age, gender, anemia status, CKD, diabetes and heart failure (Table 4). Adding RDW to model based on clinical variables, ejection fraction hemodynamic data creatinine, and MCV resulted significantly improved the model (p < 0.001) although Harrell’s-C statistic increased only slightly from 0.76 (95% CI, 0.69÷0.82) to 0.77 (95% CI, 0.70÷0.83). Adding RDW to model based on clinical, hemodynamic and laboratory parameters (including MCV) however did not change IDI significantly (IDI - 0.019 95% CI: - 0.005 ÷ 0.051, p =0.120). There was also non-significant change in the risk of death predicted by the models with and without RDW (NRI - 0.251 95% CI: - 0.163 ÷ 0.512 p = 0.213).

**Table 4:** Adjusted* hazard ratios of RDW [per 1% increase] in whole population and in subgroups

	**Hazard ratio**	**95% CI**	**P value**
Whole group	1.23	1.13-1.35	<0.0001
Females	1.31	1.06-1.62	0.01
Males	1.22	1.1-1.34	0.0001
≥75 years	1.36	1.05-1.78	0.02
<75 years	1.21	1.09-1.33	0.0003
Anemia	1.17	1.02-1.34	0.02
Without anemia	1.37	1.19-1.58	<0.0001
With CKD	1.49	1.32-1.82	0.0001
Without CKD	1.16	1.04-1.29	0.009
Diabetes	1.23	1.08-1.4	0.002
Without diabetes	1.27	1.12-1.46	0.0003
Heart failure	1.20	1.04-1.39	0.01
Without heart failure	1.39	1.07-1.8	0.01

Adjusted for age, sex, heart failure, atrial fibrillation, hypertension, previous MI, previous PCI, previous CABG, previous SCD, PVD, previous stroke, diabetes, lipid abnormalities, obesity, COPD, CKDsmoking, NYHA and CCS class, heart rate, blood pressure, ejection fraction, number and type(s) of stent implanted, number of PCI vessels, hemoglobin, MCV.

## Discussion

There are two main findings of this study. First, with increasing values of RDW, there is an increase in the rate of patients with serious comorbidities such as COPD, PVD, diabetes treated with insulin, atrial fibrillation and heart failure in the entire population. Second, the RDW was associated with mortality, even after adjusting for clinical echocardiographic and hemodynamic variables in the whole population and in various subgroups. Mortality in the patients with the highest RDW values was almost 4-fold higher than in patients with the lowest RDW values. Similar results were obtained by Fatemi O. and Tonelli M. et al., who observed a graded relationship between RDW quartiles and mortality in patients with various forms of coronary artery disease [4,11], and by Patel V.K., who reported an increase in mortality across RDW quintiles in the general population [18]. In a recent study, Ren H. et al. reported that similar trends of mortality and RDW may be present in patients with stable coronary artery disease because they registered 8 deaths (2.27%) vs. 2 deaths (0.51%) [9] in patients with RDW values in the highest and lowest RDW quartiles, respectively. Our analysis of a larger population with a longer follow-up period and 233 registered events confirms those findings.

### RDW as a marker of disease burden

An increased percentage of patients with serious comorbidities compared to patients with lower RDW values suggests that RDW is a universal marker of disease burden. Data from analyses referring to the wide spectrum of diseases seem to confirm that finding [3,18-28]. Associations between higher RDW levels and CKD are quite easy to explain because anemia, which is frequently found in patients with impaired renal function [29,30], is associated with increased RDW values. Moreover, RDW values are often used for anemia classification [31]. An explanation similar to that for CKD may hold for heart failure because Jankowska E et al. showed that iron deficiency develops during the progression of heart failure [32]. In our study, the frequency of heart failure and the percentage of patients with advanced heart failure symptoms were higher in patients with RDW values within the 4^th^ quartile. The association of RDW with other comorbidities such as COPD, diabetes and peripheral vascular disease may be more difficult to elucidate. Possible explanations come from publications describing the increased inflammatory status and increased oxidative stress in those conditions [33-38]. Therefore, in more advanced stages of atherosclerosis, COPD and diabetes, the detrimental effects of oxidative stress on erythrocyte membrane fluidity affects the lifespan of red blood cells, which in turn leads to higher RDW values [39]. However, Fornal M et al. and Lippi G et al. [40,41] reported the existence of a potential link between inflammatory biomarkers and RDW values that may also be of importance, because the inflammation may impair iron metabolism and inhibit both the production of and the response to erythropoietin [18,42]. Veranna V. et al. reported that RDW values of 12.6% and increased CRP levels above 3 mg/dl are associated with a higher risk of mortality in a cohort free of coronary heart disease [43]. Another interesting observation is the lower frequency of multivessel coronary artery disease (MVD) in patients with RDW values in the 1^st^ quartile compared to the other quartiles, which is in concordance with results of Isik T. et al. and MA F-L [44,45]. Those findings may indirectly confirm the association of increasing values of RDW with extensiveness and possibly the duration of the atherosclerotic process and the role of RDW as a marker of the disease process.

### Red blood cell heterogeneity as a potential causative factor

The association between RDW values and comorbidities does not entirely explain the increased mortality in patients with the highest RDW values because RDW remained highly significantly associated with mortality after adjusting for clinical echocardiographic, hemodynamic and laboratory parameters in the entire cohort and in subgroups, although in our study it did not improve risk prediction as estimated by NRI and IDI measures. There have been however studies showing that RDW improves prediction of bleeding after acute coronary syndromes [46] and mortality in kidney transplant recipients [47].

Currently, it is not clear why higher RDW levels are so strongly associated with worse long term prognoses in various diseases. Luneva O.G. et al. provided insight into the pathophysiology of the relationship between mortality and RDW values by finding a significant correlation between RDW values and the cholesterol content of erythrocyte membranes, which also determines erythrocyte membrane fluidity [39]. It has also been reported that greater variation in RDW is associated with impaired blood flow through the microvascular system, which may cause tissue hypoxia, even in patients without anemia [48].

### Strengths and limitations

A limitation of this study is the retrospective design. Nonetheless, the potential disadvantages of this retrospective analysis are diminished by the fact that the patient data are inputted into an electronic database from report forms filled out by the attending physician upon the patient’s admission to our center. Other limitations include a lack of iron status and other biomarkers such as high sensitivity troponins.

Strengths of this study include large cohort, detailed data on clinical, echocardiographic hemodynamic and laboratory parameters, and long follow-up period with very little patients lost to follow-up.

## Conclusions

RDW is an independent predictor of mortality in patients with stable coronary artery disease undergoing stent implantation. Higher RDW values correspond with a higher comorbidity burden and higher mortality in a stepwise fashion, even within the RDW reference range.

## Abbreviations

BMI: Body mass index; BMS: Bare metal stent; CABG: Coronary artery bypass graft; CCS: Canadian Cardiovascular Society; CHD: Coronary heart disease; CKD: Chronic kidney disease; COPD: Chronic obstructive pulmonary disease; DBP: Diastolic blood pressure; FH: Family history CHD; GFR: Glomerular filtration rate; HCT: Hematocrit; HGB: Hemoglobin; HR: Heart rate; MCV: Mean corpuscular volume; MI: Myocardial infarction; MVD: Multivessel disease; NYHA: New York Heart Association; PCI: Percutaneous coronary intervention; PDW: Platelet distribution width; PVD: Peripheral vascular disease; RBC: Red blood cells; RDW: Red cell distribution width; SBP: Systolic blood pressure; SCD: Sudden cardiac death; TIA: Transient ischemic attack; WBC: White blood cells.

## Competing interests

The authors declare that they have no competing interests.

## Authors’ contributions

TO - conception and design of the study, statistical analysis and interpretation of data, drafting the article; JS - design of the manuscript, help in drafting manuscript, interpretation of data. MH, AL, KW, KD MGa - acquisition and interpretation of data (coronary angiography records), revising the manuscript for intellectual content MGi - acquisition and interpretation of data (coronary angiography records), revising the manuscript for intellectual content. JW - design of the manuscript, interpretation and analysis of data, help in drafting the manuscript, AW - interpretation of data revising the manuscript for intellectual content. AK, ARG, AS - acquisition of data, revising the manuscript for intellectual content. LP - Design of the study, interpretation of data, revising the article for intellectual content. All authors read and approved the final manuscript.

## Pre-publication history

The pre-publication history for this paper can be accessed here:

http://www.biomedcentral.com/1471-2261/13/113/prepub
